# Prevalence and Characteristics of *Campylobacter* Throughout the Slaughter Process of Different Broiler Batches

**DOI:** 10.3389/fmicb.2018.02092

**Published:** 2018-09-04

**Authors:** Xiaoyan Zhang, Mengjun Tang, Qian Zhou, Jing Zhang, Xingxing Yang, Yushi Gao

**Affiliations:** Jiangsu Institute of Poultry Sciences, Supervision, Inspection and Testing Centre for Poultry Quality, Ministry of Agriculture, Yangzhou, China

**Keywords:** *Campylobacter*, broiler, slaughter process, prevalence, MLST, antibiotic susceptibility

## Abstract

Handling and consumption of chicken meat are risk factors for human campylobacteriosis. This study was performed to describe the *Campylobacter* population in broiler carcasses and environmental samples throughout the slaughter process. Moreover, the genetic diversity and antimicrobial resistance of the *Campylobacter* strains were evaluated. Cloacal swabs, samples from carcasses at different stages, and environmental samples were collected thrice from the different flocks at the same abattoir located in Central Jiangsu, China. *Campylobacter* isolated from the three batches (*n* = 348) were identified as *Campylobacter jejuni* (*n* = 117) and *Campylobacter coli* (*n* = 151) by multiplex PCR. Characterization by multilocus sequence typing revealed a specific genotype of *Campylobacter* for each batch. Antimicrobial sensitivity to 18 antibiotics were analyzed for all selected strains according to the agar diffusion method recommended by the Clinical and Laboratory Standards Institute. Antibiotic susceptibility tests indicated that the majority of the tested isolates were resistant to quinolones (>89.7%). Less resistance to macrolide (59.8%), gentamicin (42.7%), amikacin (36.8%) was observed. Results showed that 94.0% of the tested strains demonstrated multidrug resistance.

## Introduction

*Campylobacter* is a leading cause of bacterial foodborne infections in developed countries. This infection has surpassed *Salmonella* several years ago and caused a significant economic burden (EFSA and ECDC, 2016). Although new species of *Campylobacter* have recently been discovered, human campylobacteriosis are dominated by two main species, *Campylobacter jejuni* and *Campylobacter coli* (Tresse et al., 2017). *Campylobacter* infection causes watery diarrhea, abortion, human acute enteritis, and several complications, such as Guillain-Barré syndrome and Reiter’s syndrome, in severe cases. Handling and consumption of poultry are the major sources for human infection (Boysen et al., 2014). Reducing the prevalence or number of *Campylobacter* in broilers at the primary stage could be an effective way to protect public health from *Campylobacter* infections (European Food Safety Authority [EFSA], 2010). However, despite the many biosecurity interventions at the farm, *Campylobacter* has not been well controlled in broiler flocks after the rearing period (Newell et al., 2011).

During slaughter, many opportunities may facilitate cross-contamination and spread of bacteria despite good hygiene (Althaus et al., 2017). Poultry meat from *Campylobacter*-negative flocks may be contaminated by previously slaughtered *Campylobacter*-positive flocks (Miwa et al., 2003; Allen et al., 2007). *Campylobacter* can be spread to the poultry meat in the slaughter line, especially after evisceration or from dirty surfaces (Corry and Atabay, 2001; Melero et al., 2012). The prevalence of *Campylobacter* within positive flocks at slaughter are high (approximately 80%) (Colles et al., 2010; European Food Safety Authority [EFSA], 2010). However, in China, few studies have been conducted on the contamination of broiler carcasses throughout the production chain. During slaughter, *Campylobacter* could also be recovered in the processing equipment and environmental samples (Berrang et al., 2000; Cason et al., 2007; Ellerbroek et al., 2010). Studies have shown that some *Campylobacter* strains recovered from the slaughterhouse environment can contaminate carcasses when several batches of poultry are slaughtered (Peyrat et al., 2008; Melero et al., 2012). Previous studies only assessed the slaughtering performance to identify operations that increase or decrease the contamination of carcasses (Habib et al., 2012; Seliwiorstow et al., 2015). Moreover, the possibility of *Campylobacter* in plants as a continuous source of contamination is still ambiguous (García-Sánchez et al., 2017).

*Campylobacter* outbreaks are sporadic and caused by cross-contamination, and these characteristics hamper the determination of the sources of contamination. Molecular methods play an important role in the epidemiological study of tracing sources and routing of pathogen transmission. Pulsed-field gel electrophoresis and multilocus sequence typing (MLST) have been employed successfully for the epidemiological study of *Campylobacter* from different sources and outbreaks (Dingle et al., 2005; Michaud et al., 2005; Thakur et al., 2009). The resistance of *Campylobacter* to antibiotics has also been a persistent issue generally related to the indiscriminate use of antibiotics for therapy or as a growth promoter (Chen et al., 2010; Rozynek et al., 2013). Determining the drug resistance of *Campylobacter* strains is important to control and prevent human infection. In China, molecular subtyping and antimicrobial susceptibilities of *Campylobacter* strains from different sources have been conducted (Zhang et al., 2014; Zeng et al., 2016). However, limited data are available on the comprehensive molecular characterization and antibiotic susceptibility of *Campylobacter* from broilers during slaughter.

This study was performed to determine the prevalence of *Campylobacter* in the slaughter and poultry processing environment. Moreover, the genotype characteristics and antibiotic susceptibility of these strains were assessed.

## Materials and Methods

### Sample Collection

Samples were collected from a slaughterhouse located in Central Jiangsu, China. This slaughterhouse processes 20,000 broilers per day. We selected three batches (birds from one flock slaughtered at the same day) in different farms from April 2017 to November 2017. During each visit, cloacal samples were individually collected using sterile swabs before slaughter. Then, samples from the broiler carcasses were collected after plucking, evisceration, washing, and chilling. Water from the cleaning pool and swabs from the operating table and workers’ gloves were also collected as environmental samples. Furthermore, sample collection was performed consecutively during 1 h of the slaughter process. All cloacal swabs were placed in Cary-Blair medium. Swab samples taken at different points were collected using cotton swabs moistened with sterile saline and stored in aseptic bags. Water samples were placed in sterile plastic containers. All samples were transported to the laboratory under cool conditions within 3 h and analyzed the same day. The number of samples collected at different points are shown in **Table 1**.

**Table 1:** Contamination ratio of *Campylobacter* during slaughter process in three batches.

Sample site	No. of *Campylobacter* positive samples /total no. of samples (%)					
	Batch 1 (Farm 1)		Batch 2 (Farm 2)		Batch 3 (Farm 3)	
	*C. jejuni*	*C.coli*	*C. jejuni*	*C. coli*	*C. jejuni*	*C. coli*
Cloacal swabs	19/30 (63.3%)	0	0	20/20 (100.0%)	0	3/22 (13.6%)
After plucking	22/30 (73.3%)	0	0	18/21 (85.7%)	4/15 (26.7%)	10/15 (66.7%)
After evisceration	24/30 (80.0%)	0	0	17/22 (77.3%)	0	12/15 (80.0%)
After washing	23/30 (76.7%)	0	0	20/21 (95.2%)	3/20 (15.0%)	11/20 (55.0%)
After chilling	7/8 (87.5%)	0	1/10 (10.0%)	9/10 (90.0%)	0	10/10 (100.0%)
Operating table	4/5 (80.0%)	0	0	5/5 (100.0%)	1/10 (10.0%)	9/10 (90.0%)
Workers’ gloves	3/5 (60.0%)	0	1/5 (20.0%)	4/5 (80.0%)	2/5 (40.0%)	2/5 (40.0%)
Water	3/3 (100.0%)	0	0	1/3 (33.3%)	0	0/3/0
Total	105/141 (74.5%)		96/107 (89.7%)		67/100 (67.0%)	

### *Campylobacter* Isolation and Identification

All cloacal swab samples were placed in 1 mL of PBS (phosphate buffer saline) for full immersion. Then, the swabs were removed from the solution. After 10 times dilution, 100 μL of each solution was placed on modified charcoal-cefoperazone-deoxycholate agar (mCCDA) (Oxoid CM0739) with cefoperazone (LKT, C1630), amphotericin B (Wako, 011-1363), and rifampicin (Wako, 185-01003). All plates were incubated at 42°C for 36–42 h in a microaerophilic environment (5% O_2_, 10% CO_2_, and 85% N_2_).

Cotton swabs from carcasses and environment were enriched in 10 mL of Bolton Broth (BB, CM0983, Oxoid; supplemented with BB Selective Supplement, SR0183E, Oxoid) based on previous reports (Adzitey et al., 2012; Tissier et al., 2012). Water from the cleaning pool were filtered through filters with a 0.22 μm pore diameter. Then the membranes were introduced into 10 mL of BB. After 24 h of incubation at 42°C in glass jars under a microaerobic atmosphere, 10 μL of the resulting solutions were streaked onto mCCDA plates, and the plates were incubated at 42°C for 48 h.

After incubation, the suspected colonies were picked and subcultured in Mueller–Hinton (MH) agar (Difco, MD) with blood and incubated for 24–48 h at 42°C under microaerobic conditions. Multiplex PCR tests were used to confirm and identify whether the strains were *Campylobacter jejuni* or *Campylobacter coli* according to a previous study (Huang et al., 2009).

### Antibiotic Susceptibility Testing

Susceptibility of *Campylobacter* to 18 antibiotics (Oxoid) from six classes of antibiotics was determined using the following antimicrobial impregnated disks (Oxoid, England, United Kingdom): β-lactams (ampicillin, AMP, 10 μg; amoxicillin, AML, 30 μg; cefotaxime, CEX, 10 μg; and ceftriaxone, CRO, 30 μg); aminoglycosides (streptomycin, S, 10 μg; gentamicin, GEN, 10 μg; kanamycin, K, 30 μg; amikacin, AMK, 30 μg; and tobramycin, TOB, 10 μg); quinolones (norfloxacin, NOR, 10 μg; ciprofloxacin, CIP, 5 μg; ofloxacin, OFX, 5 μg; nalidixic acid, NA, 30 μg; and enrofloxacin, ENR, 5 μg); macrolide (erythromycin, E, 15 μg; and azithromycin, AZM, 15 μg); tetracycline (TE, 30 μg); and clindamycin (DA, 2 μg). This process was carried out according to the Kirby-Bauer disk diffusion method (Bauer et al., 1966) and as recommended by the Clinical Laboratory Standards Institute (Clinical and Laboratory Standards Institute, 2012). Some isolates were revived from the glycerol stocks using BB with 10% lysed sheep blood. Then, these isolates were incubated for 36 h at 42°C under microaerobic conditions. Revived cultures were streaked using sterile cotton swabs on MH (Mueller Hinton) agar plates (Oxoid) supplemented with 10% sheep blood for another 36 h of incubation. The bacteria were scrapped to PBS, and the turbidity of the suspension was adjusted to 0.5 McFarland standard (Clinical and Laboratory Standards Institute, 2012). The suspension was stacked onto MH agar plates for 36 h of incubation under microaerophilic conditions. *Escherichia coli* ATCC 25922 and *C. jejuni* NCTC 11168 were used as reference strains.

The diameters of the inhibition zones around the antibiotic disk were measured. The breakpoints used to categorize isolates as susceptible, intermediate, and resistant–were based on the Clinical and Laboratory Standards Institute recommendations (Clinical and Laboratory Standards Institute, 2012). Isolates resistant to ≥3 unrelated antibiotic classes were classified as isolates with multidrug resistance (MDR).

MAR (Multiple Antimicrobial Resistance) was used to quantify the multi-resistance of *Campylobacter* isolates. MAR index = a/b. In this formula, “a” indicated the number of antibiotics to which the isolate was resistant and “b” indicated the total number of antibiotics to which the isolate was tested (Krumperman, 1983).

### Multilocus Sequence Typing for *Campylobacter*

DNA was extracted from some representative strains using a commercial DNA Kit (Tiangen Biotech Inc., Beijing, China). MLST was conducted using primer sequences obtained from http://pubmlst.org/ *Campylobacter* as previously described (Dingle et al., 2001). The nucleotide sequences of the amplicons were determined by GenScript, Inc. (Nanjing, China). Allele numbers and STs (sequence types) were assigned using the *Campylobacter* PubMLST database.

### Data Analysis

The difference in the prevalence levels across the batches in cloacal swabs and various sample points obtained after plucking, evisceration, washing and chilling were analyzed using a nonparametric test (Chi-square test) using SPSS software (version 17.0). *P* < 0.05 were considered statistically significant.

Sequence analysis and ST determinations of clonal complexes were performed using the PubMLST database^1^ for ST designation. Consensus tree was constructed using UPGMA cluster analysis based on the seven housekeeping gene sequences.

## Results

### Prevalence of *Campylobacter* in the Slaughter Process

The isolation rates of *Campylobacter* and the samples collected from each point are listed in **Table 1**. All strains isolated in batch 1 were *C. jejuni* (105/105). However, in batches 2 and 3, *C. coli* was the predominant isolated strain (94/96 and 57/67, respectively). The contamination rate of *Campylobacter* on the carcasses at every point during slaughter was relatively high even after chilling (87.5–100%). As shown in **Table 2**, compared with batches 1 and 2, *Campylobacter* infection rate in the cloacal swabs in batch 3 was significantly lower (*P* < 0.05). However, significant correlations of infection rates were not observed after evisceration among different batches.

**Table 2:** The statistical contrast across the three batches (*P*-value).

Sample site	Batch (Farm) (No. of *Campylobacter* positive samples/total no. of samples)		*P*-value
Cloacal swabs	1 (19/30)	2 (20/20)	0.317
	1 (19/30)	3 (3/22)	0.000^∗^
	2 (20/20)	3 (3/22)	0.000^∗^
After plucking	1 (22/30)	2 (18/21)	0.295
	1 (22/30)	3 (14/15)	0.118
	2 (18/21)	3 (14/15)	0.480
After evisceration	1 (24/30)	2 (17/22)	0.814
	1 (24/30)	3 (12/15)	1.000
	2 (17/22)	3 (12/15)	0.845
After washing	1 (23/30)	2 (20/21)	0.076
	1 (23/30)	3 (14/20)	0.602
	2 (20/21)	3 (14/20)	0.034^∗^
After chilling	1 (7/8)	2 (10/10)	0.264
	1 (7/8)	3 (10/10)	0.264
	2 (10/10)	3 (10/10)	N

*“N” means no statistics are computed; “^∗^” resembles P < 0.05.*

At the batch level, we compared the contamination rates of *Campylobacter* at four sampling points (after plucking, evisceration, washing, and chilling) and found no significant difference except the point of after washing between batches 2 and 3 (*P* = 0.034) (**Table 2**). A total of 268 isolates were obtained from 348 samples (77.0%), including 117 *C. jejuni* and 151 *C. coli* isolates.

*Campylobacter* isolates were recovered from all environmental samples. The cotton swab samples from the operating table and workers’ gloves in the 3 batches showed relatively high contamination levels. However, *Campylobacter* species were not isolated from the cooled water in batch 3.

### Antimicrobial Resistance

We selected 39 isolates (*C. jejuni*) from batch 1, 38 isolates (2 *C. jejuni* and 36 *C. coli*) from batch 2, and 40 isolates (1 *C. jejuni* and 39 *C. coli*) from batch 3 for antimicrobial resistance testing. A total of 18 antimicrobials classified under six antimicrobial groups were employed to test the selected *Campylobacter* isolates. Except for seven samples from batch 2, all isolates were resistant to at least one or more antimicrobials (**Table 4**). The majority of the tested isolates were resistant to quinolones (≥89.7%). Less resistance to macrolide (59.8%), gentamicin (42.7%), and amikacin (36.8%) was observed. The resistance to other antibiotics in this study was greater or equal to 65.0%. Resistance to macrolide was not detected from batch 1, but 81.6 and 100% from batches 2 and 3, respectively, were resistant to this antimicrobial (**Table 3**).

**Table 3:** Number and percentages of resistance of *Campylobacter* isolates from three batches.

Antibiotic group	Antibiotic name	No. of resistant *Campylobacter* isolates (%)			
		Batch 1	Batch 2	Batch 3	Total
β-Lactams	AMP	39/39 (100%)	31/38 (81.6%)	7/40 (17.5%)	77/117 (65.8%)
	AML	39/39 (100%)	31/38 (81.6%)	7/40 (17.5%)	77/117 (65.8%)
	CEX	39/39 (100%)	34/38 (89.5%)	40/40 (100%)	113/117 (96.6%)
	CRO	39/39 (100%)	20/38 (52.6%)	40/40 (100%)	99/117 (84.6%)
Aminoglycosides	S	39/39 (100%)	31/38 (81.6%)	40/40 (100%)	109/117 (93.2%)
	GEN	39/39 (100%)	5/38 (13.2%)	6/40 (15.0%)	50/117 (42.7%)
	K	39/39 (100%)	31/38 (81.6%)	40/40 (100%)	109/117 (93.2%)
	AMK	39/39 (100%)	2/38 (5.7%)	2/40 (5.0%)	42/117 (36.8%)
	TOB	39/39 (100%)	15/38 (39.5)	40/40 (100%)	94/117 (80.3%)
Quinolones	NOR	39/39 (100%)	31/38 (81.6%)	40/40 (100%)	109/117 (93.2%)
	CIP	39/39 (100%)	31/38 (81.6%)	40/40 (100%)	109/117 (93.2%)
	OFX	39/39 (100%)	31/38 (81.6%)	40/40 (100%)	109/117 (93.2%)
	NA	39/39 (100%)	31/38 (81.6%)	40/40 (100%)	109/117 (93.2%)
	ENR	39/39 (100%)	26/38 (68.4)	40/40 (100%)	105/117 (89.7%)
Macrolide	E	0	31/38 (81.6%)	40/40 (100%)	70/117 (59.8%)
	AZM	0	31/38 (81.6%)	40/40 (100%)	70/117 (59.8%)
Tetracyclines	TE	39/39 (100%)	31/38 (81.6%)	40/40 (100%)	109/117 (93.2%)
Clindamycin	DA	39/39 (100%)	31/38 (81.6%)	37/40 (92.5%)	106/117 (90.6%)

The MAR (Multiple Antimicrobial Resistance) indices of the tested isolates from the current study are indicated in **Table 4**. MDR (resistance to three or more antimicrobial families) was observed in the majority of the isolates. A total of 17 different antibiotic resistance patterns with MAR index ranging from 0 to 1.00 were observed. Multiple resistances were common with resistance from 14 to 16 to 18 antibiotics (MAR index 0.78–0.89). This characteristic was observed in most of the 117 Campylobacter strains. Strains from batch 1 showed resistance to 16 antibiotics (except AZM and E). The resistance spectra of the strains from batches 2 and 3 were more diverse than that of the strains from batch 1. A total of 94.0% of the tested strains demonstrated MDR.

**Table 4:** Resistance spectra of 117 *Campylobacter* to various antibiotic combinations.

MAR index	No. of *Campylobacter* isolates			Antibiotic
	Batch 1	Batch 2	Batch 3	
0	0	7	0	–
0.72	0	1	0	AMP, AML, CEX, CRO, S, K, NOR, CIP, NA, E, AZM, TE, DA
0.78	0	2	0	AMP, AML, CEX, CRO, S, K, NOR, CIP, OFX, NA, E, AZM, TE, DA
0.78	0	2	0	AMP, AML, CEX, S, K, NOR, CIP, OFX, NA, ENR, E, AZM, TE, DA
0.78	0	0	33	CEX, CRO, S, K, TOB, NOR, CIP, OFX, NA, ENR, E, AZM, TE, DA
0.83	0	5	0	AMP, AML, CEX, S, K, TOB, NOR, CIP, OFX, NA, ENR, E, AZM, TE, DA
0.83	0	2	0	AMP, AML, CEX, CRO, S, K, TOB, NOR, CIP, NA, ENR, E, AZM, TE, DA
0.83	0	4	0	AMP, AML, CEX, CRO, S, K, NOR, CIP, OFX, NA, ENR, E, AZM, TE, DA
0.83	0	0	2	AMP, AML, CEX, S, GEN, K, TOB, NOR, CIP, OFX, NA, ENR, E, AZM, TE,
0.83	0	1	0	AMP, AML, CEX, S, GEN, K, TOB, NOR, CIP, OFX, NA, E, AZM, TE, DA
0.89	0	9	0	AMP, AML, CEX, CRO, S, K, TOB, NOR, CIP, OFX, NA, ENR, E, AZM, TE, DA
0.89	0	1	0	AMP, AML, CEX, GEN, K, AMK, TOB, NOR, CIP, OFX, NA, ENR, E, AZM, TE, DA
0.89	0	1	0	CEX, CRO, S, GEN, K, AMK, TOB, NOR, CIP, OFX, NA, ENR, E, AZM, TE, DA
0.89	0	0	2	AMP, AML, CEX, CRO, S, GEN, K, TOB, NOR, CIP, OFX, NA, ENR, E, AZM, TE
0.89	39	0	0	AMP, AML, CEX, CRO, S, GEN, K, AMK, TOB, NOR, CIP, OFX, NA, ENR, TE, DA
0.94	0	3	1	AMP, AML, CEX, CRO, S, GEN, K, TOB, NOR, CIP, OFX, NA, ENR, E, AZM, TE, DA
1.00	0	0	2	AMP, AML, CEX, CRO, S, GEN, K, AMK, TOB, NOR, CIP, OFX, NA, ENR, E, AZM, TE, DA

### MLST Analysis of *Campylobacter* Isolated From the Slaughter Process and the Environment

Isolates from cloacal swabs, environmental samples, and carcasses at different slaughter points were selected and subjected to MLST analysis. The STs, species, sources, and numbers of bacteria are summarized in **Table 5**. A total of eight STs, including one novel type, were observed from the 117 isolates. Three *C. jejuni* STs and 7 *C. coli* STs were identified. One clonal complex CC828 (55 isolates) was generated from these isolates, but 62 isolates could not be assigned to any of the defined CCs.

**Table 5:** Distributions of STs for 117 *Campylobacter* isolates.

Batch	species	ST	ST-CC	Source	Number
1	*C. jejuni*	8089	UA	Cloacal swab	7
		8089	UA	After plucking	7
		8089	UA	After evisceration	8
		8089	UA	After washing	4
		8089	UA	After chilling	7
		8089	UA	Operating table	2
		8089	UA	Workers’ gloves	2
		8089	UA	Water	2
2	*C. jejuni*	6186	UA	After chilling	1
	*C. coli*	6186	UA	Cloacal swab	3
		5511	828	Cloacal swab	1
		6186	UA	After plucking	6
		6186	UA	After evisceration	3
		5511	828	After washing	1
		825	828	After washing	1
		6186	UA	After washing	4
		NEW1	–	After washing	2
		825	828	After chilling	2
		860	828	After chilling	3
		6186	UA	After chilling	3
		872	828	Operating table	2
		860	828	Operating table	1
		872	828	Workers’ gloves	4
		6186	UA	Water	1
3	*C. jejuni*	860	828	Workers’ gloves	2
	*C. coli*	860	828	Cloacal swab	3
		860	828	After plucking	6
		830	828	After plucking	1
		860	828	After evisceration	6
		860	828	After washing	5
		6186	828	After washing	1
		860	828	After chilling	7
		830	828	After chilling	1
		825	828	After chilling	1
		860	828	Operating table	4
		825	828	Operating table	2
		860	828	Workers’ gloves	1

*“ST-CC” means ST clonal complex; “UA” means unassigned.*

**FIGURE 1:**
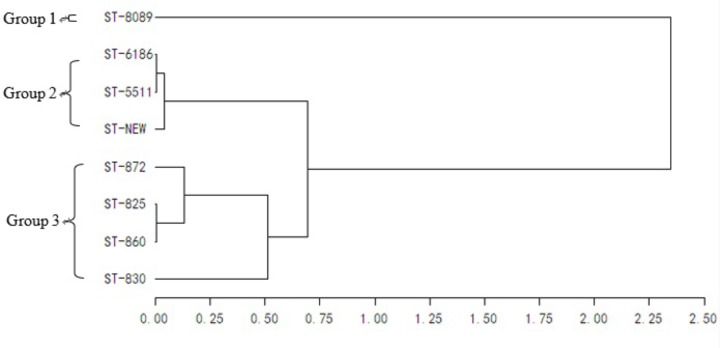
Genetic relationships of the isolates based on the MLST. The consensus tree was developed using UPGMA cluster analysis.

All 39 *C. jejuni* isolates from batch 1 were identified as ST8089. Compared with batch 1, batches 2 and 3 showed more diversity in STs. In batches 2 and 3, ST6186 (21/38, 55.3%) and ST860 (33/40, 82.5%) were the most frequently observed STs. This result was similar to the isolates from each cloacal sample. In addition, the STs from the environmental samples in each batch were highly consistent with those from the carcasses and cloacal swabs. In batch 2, strains after washing contained three traditional and one new ST types. All identified STs were further analyzed using UPGMA (**Figure 1**). Eight identified STs were classified into three clonal groups. All *C. jejuni* isolates from batch 1 belonged to group 1, and most of the *Campylobacter* isolates in batches 2 and 3 belonged to groups 2 and 3, respectively.

## Discussion

*Campylobacter* infection is considered one of the leading causes of bacterial gastroenteritis in developed and developing countries (Wei et al., 2014; Kaakoushm et al., 2015; Nohra et al., 2016). Several studies have associated the risk of human *Campylobacter* infection with highly contaminated broiler carcasses (Callicott et al., 2008; Nauta and Havelaar, 2008). Although China is one of the largest poultry producers worldwide, data on this pathogen are limited, especially during broiler production. In the present study, we showed the contamination rate of *Campylobacter* in samples collected along the slaughter line, that is, from the cloacal swabs in live birds to post chiller carcasses. The prevalence of *Campylobacter* after chilling was high (87.5–100%). This result was consistent with the finding of Seliwiorstow and collaborators, who compared the carcass contaminations before washing and after chilling in the slaughterhouse and found that the contamination rates were not reduced (Seliwiorstow et al., 2015). Accordingly, improved hygiene during slaughtering may reduce the number of *Campylobacter* in the carcasses, but the infection rates cannot be reduced because of cross-contamination. Despite the significantly low *Campylobacter* infection rate of cloacal swabs in batch 3 (*P* < 0.05), significant correlations of infection rates after evisceration among the different batches were not observed. These data explained that in the case of batch 3, which had lower positive cloacal numbers, the cross-contamination coming from the intestinal content of other flocks or its own flock still existed.

The investigation was conducted in 2012 in a commercial poultry production in Shanghai, China. A total of 23 *C. jejuni* (17.0%) and 33 *C. coli* (24.4%) were isolated from 135 broiler carcasses from the slaughterhouse (Ma et al., 2014). Wang and collaborators investigated the prevalence of *Campylobacter* from broiler chickens in slaughter houses in five Chinese provinces during 2008–2014, and isolated 977 *C. jejuni* (18.1%) and 1021 *C. coli* (19.0%) from 5,385 chickens (Wang et al., 2016). In southern Brazil, samples from the broiler slaughtering process were analyzed to directly count *Campylobacter* and results showed that 72 and 38% were *C. jejuni* and *C. coli*, respectively (Gonsalves et al., 2016). Similar results were demonstrated in the European Union, where 60.8% of broiler samples tested positive for *C. jejuni*, and 41.5% tested positive for *C. coli* (European Food Safety Authority [EFSA], 2010). In the present study, a total of 117 *C. jejuni* (33.6%) and 151 *C. coli* (43.4%) were isolated from 348 samples collected in the slaughterhouse. The isolation rates were higher than those obtained in previous years in China. More samples are needed for further study of the prevalence of *Campylobacter* species found in the different steps of the slaughter process.

*Campylobacter* isolates were recovered from all environmental samples. *Campylobacter* can form biofilms to survive outside the host and protect against chemical products, physical cleaning processes, and environmental stress, and these processes are proposed as a survival mechanism (Hanning et al., 2008). Thus, this mechanism may explain the presence of *Campylobacter* in the cool water despite the chemical treatment of the water. Defeathering and evisceration are considered as critical contamination steps in poultry processing (Sasaki et al., 2013; Gruntar et al., 2015). Samples from the operating table and workers’ gloves showed a high contamination level in this study. This result suggested an important cross-contamination rate between carcasses and processing equipment. Therefore, it is recommended that contaminated broiler flocks should be slaughtered at the end of the working day to reduce the cross-contamination among the flocks.

Antibiotic resistance is a persistent issue in veterinary medicine and human medical treatment because of the indiscriminate use of antibiotics in therapy or as a growth promoter. Although most *Campylobacter* infections are self-limiting and do not require any antibiotic treatment, antimicrobial treatment is necessary for some severe and prolonged cases. Fluoroquinolones and macrolides are usually administered to treat human campylobacteriosis in China (Zhang et al., 2014). Our results showed that more than 89.7% of the tested isolates were resistant to quinolones and 59.8% of the tested isolates were resistant to macrolide. This result was consistent with those of previous reports (Li et al., 2018). All tested isolates of *C. jejuni* from batch 1 were susceptible to macrolide (AZM and E), compare with 81.6 and 100% of the tested isolates of *C. coli* from batches 2 and 3, respectively, resistant to this antimicrobial. These data were in accordance with recent reports (Ruzauskas et al., 2011; Haruna et al., 2012; Fraqueza et al., 2014; Pergola et al., 2017), which indicated that *C. jejuni* was predominantly susceptible to erythromycin while *C. coli* was resistant. However, another possible reason for the results in the current study is that *Campylobacter* isolated from the same batch may have primarily the same antibiotic resistance profile.

MDR was observed in the majority of the tested isolates (94%) in this study. Higher frequency of MDR was also noted in *C. coli* isolates from different sources (99%) in China (Zhang et al., 2014). In the present study, 17 different antibiotic resistance patterns with MAR index ranging from 0 to 1.00 were observed. The majority of the MAR index calculated ranged from 0.78 to 0.89 among the 117 selected *Campylobacter* strains. Strains with a MAR index >0.2 have been identified from animals frequently treated with antimicrobials (Marian et al., 2012). In contrast, significantly lower resistance rates of ciprofloxacin were observed in *Campylobacter* from poultry meat in countries with strict antimicrobial controls (Miflin et al., 2007; Zhao et al., 2010). Hence, severe MDR, which may be a threat to public safety in China, should be given proper attention.

*Campylobacter* isolates from one farm showed primarily the same genotype and the same antibiotic resistance profile as previously reported (Pergola et al., 2017). Some studies have supported the hypothesis that the contamination of *Campylobacter* in broiler carcasses is mainly from the processed *Campylobacter*-positive birds within a batch (Rasschaert et al., 2006; Sasaki et al., 2014). In the present study, the composition of the MLST was relatively stable within a batch because of the predominance of certain MLST types. In each batch, the most frequently observed STs (ST8089, ST6186, and ST860) were similar to the STs of the isolates from each cloacal sample. **Figure 1** shows the close genetic relationship with the dominant STs in each batch. These results may indicate that *Campylobacter* in slaughterhouses originated mainly from the farms. Thus, minimizing the *Campylobacter* colonization in the incoming broiler flock is important to reduce the public health risk.

Several isolates collected in this study shared identical genotypes (ST6186, ST825, ST830, ST860, and ST872) with those isolates from the feces of a diarrheal patient in China (Zhang et al., 2014). The consistency of STs in the environmental and carcass samples suggested that some environmental samples, such as those from the operating table and workers’ gloves, may reflect the potential source of contamination. This result further indicated the importance of good hygienic practices during the slaughter process.

## Author Contributions

QZ, JZ, and XY performed the collection of samples. XZ and QZ did the *Campylobacter* detection and identification. MT and XZ performed the MLST and antibiotic susceptibility tests. QZ and MT did the data analysis. XZ prepared the manuscript. YG supervised and assisted in the manuscript preparation.

## Conflict of Interest Statement

The authors declare that the research was conducted in the absence of any commercial or financial relationships that could be construed as a potential conflict of interest.

## Footnotes

1 http://pubmlst.org/campylobacter/
